# The Optimal Timing for Initiating Anamorelin in the Treatment of Cancer Cachexia

**DOI:** 10.7759/cureus.81622

**Published:** 2025-04-02

**Authors:** Miki Ogura, Hiroshi Matsuoka, Saeri Shinohara, Yusuke Umeki, Noriaki Mastumoto, Tomohiro Mizuno, Masanobu Usui, Yoshiki Hirooka, Kazuyoshi Imaizumi, Koichi Suda

**Affiliations:** 1 Department of Food and Nutrition Service, Fujita Health University Hospital, Toyoake, JPN; 2 Department of Surgery, Fujita Health University, Toyoake, JPN; 3 Department of Pharmacotherapeutics and Informatics, Fujita Health University, Toyoake, JPN; 4 Department of Surgery and Palliative Medicine, Fujita Health University, Toyoake, JPN; 5 Department of Gastroenterology and Hepatology, Fujita Health University, Toyoake, JPN; 6 Department of Respiratory Medicine, Fujita Health University, Toyoake, JPN

**Keywords:** anamorelin, anorexia, cancer cachexia, nutrition, refractory cachexia

## Abstract

Objective

In 2021, anamorelin, an orally active ghrelin receptor selective antagonist, was approved for the treatment of cachexia in patients with non-small cell lung cancer, gastric cancer, pancreatic cancer, and colon cancer. Cancer cachexia is classified into three stages: pre-cachexia, cachexia, and refractory cachexia, with the pre-cachexia and cachexia stages considered reversible with a combination of nutritional therapy, pharmacotherapy, and exercise therapy. In addition, treatment of cachexia requires early intervention, but diagnosis and early detection of cachexia are difficult. We hypothesized that the initiation of anamorelin treatment may be delayed in clinical practice and explored the appropriate timing of treatment initiation.

Methods

The data of patients with cachexia who received anamorelin at our hospital from June 2021 to July 2023 were retrospectively reviewed. Anamorelin was administered to 201 patients, of whom 134 were included in the study. Survival time and duration of medication were compared based on the number of objective criteria for anamorelin prescription (C-reactive protein [CRP] >0.5 mg/dL, hemoglobin <12 g/dL, albumin <3.2 g/dL). Multivariate analysis was used to determine the factors associated with continuation of anamorelin treatment for 12 weeks.

Results

Patients with a higher number of objective criteria for anamorelin prescription (CRP >0.5 mg/dL, hemoglobin <12 g/dL, albumin <3.2 g/dL) had shorter anamorelin treatment duration and survival. In multivariate analysis, 12 weeks of anamorelin treatment was associated with CRP. Comparing CRP ≤0.5 mg/dL vs. CRP >0.5 mg/dL, survival was significantly longer for CRP ≤0.5 mg/dL (p < 0.01).

Conclusions

Initiating anamorelin treatment with close attention to CRP and ensuring that prescribing criteria are met may be helpful in treating cachexia.

## Introduction

Cancer cachexia is a complex, multifaceted syndrome defined by a decrease in skeletal muscle mass caused by various combinations of reduced dietary intake and metabolic abnormalities not reversible by nutritional therapy [1]. Cachexia occurs in 50-80% of cancer patients and accounts for 20% of cancer-related deaths [2]. Cancer cachexia is also associated with chemotherapy toxicity, which decreases prognosis and quality of life [3-5]. Anamorelin, an orally active ghrelin receptor selective antagonist, has been shown to increase lean body mass and is associated with improved appetite in non-small cell lung cancer patients after 12 weeks of treatment [6]. It has also been shown to be effective in gastrointestinal cancers [7] and was approved in Japan in 2021 for the treatment of cachexia in patients with non-small cell lung cancer, gastric cancer, pancreatic cancer, and colorectal cancer. While approximately 60% of patients in these clinical trials were able to complete 12 weeks of anamorelin dosing, only about 30% of patients in the interim anamorelin postmarketing surveillance (PMS) reports were taking the drug for more than 65 to 85 days (7.2 to 12.1 weeks). In clinical practice, only a few patients were taking the drug beyond the 12 weeks of the clinical trial. Cancer cachexia is classified into three stages: pre-cachexia, cachexia, and refractory cachexia, and is considered reversible when combined with nutritional therapy, drug therapy, and exercise therapy in the pre-cachexia and cachexia stages, and the need for early intervention is reported [1]. One of the characteristics of refractory cachexia is resistance to antitumor therapy. Patients with refractory cachexia with no drug treatment at the start of anamorelin use and no future drug therapy planned were approximately 20% in the clinical trial [6,7] and 30% in the PMS interim report. Based on these reports, we hypothesized that the difficulty in diagnosing cachexia may have delayed the initiation of anamorelin therapy in clinical practice and explored the appropriate timing of treatment initiation.

## Materials and methods

Patients and survey

Patient background at the time of initiation of anamorelin treatment was retrospectively reviewed using electronic medical records. Diagnosis and treatment were made in accordance with the guidelines for the cancer type. From June 2021 to July 2023, 201 patients who met the prescribing criteria for anamorelin (patients who had experienced at least 5% weight loss and anorexia within the past six months and had at least two of the following symptoms: (1) fatigue or malaise; (2) generalized muscle weakness; (3) one or more of the following: C-reactive protein (CRP) >0.5 mg/dL, hemoglobin <12 g/dL, or albumin <3.2 g/dL) and were treated with anamorelin were included in the study. Sixteen patients who received one-time prescriptions with no confirmed dosing and 51 patients with missing data at the beginning of the anamorelin treatment were excluded from the study. The study was conducted in accordance with the Declaration of Helsinki and was approved by the Ethics Committee of our institution (No. HM23-467). Informed consent was obtained using the opt-out method, in which information about the study was provided to the participants, and they were given the opportunity to decline participation. Those who did not opt out were considered to have consented to participate. Survey items included sex, age, cancer type, chemotherapy, medical narcotics, body mass index, hemoglobin, albumin, CRP, number of objective prescription criteria (CRP >0.5 mg/dL, hemoglobin <12 g/dL, albumin <3.2 g/dL), Glasgow prognostic score (GPS), and Onodera’s prognostic nutritional index (PNI) [8]. PNI was calculated as 10 x serum albumin (g/dL) + 0.005 x lymphocyte count (/mm^3^). GPS classification was 2 for patients with both elevated CRP (1.0 mg/dL) and hypoalbuminemia (3.5 g/dL), 1 for patients with only one of these abnormalities, and 0 for patients with neither. In this study, patients were followed for 365 days from the start of anamorelin treatment to the date of death or until death.

Statistical analyses

Continuous variables were expressed as median and interquartile range and compared using the Mann-Whitney U test. Categorical variables were analyzed using the chi-square test. The Cochran-Armitage test was used for multiple groups. Duration of treatment and survival were analyzed by the Kaplan-Meier method with the log-rank test. Because body weight and skeletal muscle mass have been reported to increase after 12 weeks of anamorelin treatment [6,7], logistic regression analysis was performed to explore the factors associated with the anamorelin treatment duration of less than 12 weeks. The presence of continued anamorelin treatment for more than 12 weeks was used as the dependent factor, and hemoglobin, albumin, and CRP, the objective indicators at the start of anamorelin prescription, were used as explanatory variables. P-values <0.05 were considered significant. The aforementioned statistical analyses were performed using JMP ver. 10 (SAS Institute, Tokyo, Japan).

## Results

Patient background

**Figure 1:**
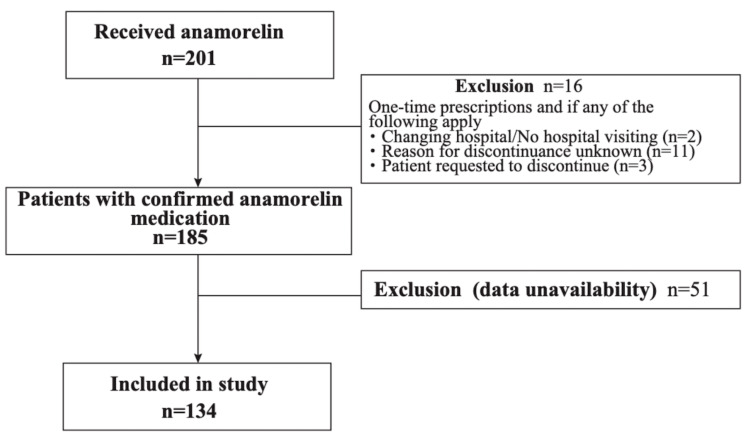
Study flow

Anamorelin was administered to 201 patients, of whom 134 were included in the study (Figure 1). The background characteristics of the subject patients at the time of anamorelin administration are shown in Table 1. The subjects' median (interquartile range) for age, body mass index, hemoglobin, albumin, and CRP were 71 (64-78) years, 18.9 (16.8-21.3) kg/m^2^, 10.5 (9.2-12.2) g/dL, 3.2 (2.8-3.5) g/dL, 1.35 (0.24-4.63) mg/dL, respectively. Sixty-four percent of patients received chemotherapy, and 52.2% fell into GPS category 2.

**Table 1:** Patients' background characteristics IQR: interquartile range.

Characteristic	All (N = 134); n (%), median ＜IQR＞
Sex	
Male	86 (64.2)
Female	48 (35.8)
Age	71 <64-78>
Primary site of cancer	
Gastric	32 (23.9)
Colorectum	24 (17.9)
Pancreas	28 (20.9)
Lung (non-small cell lung cancer)	50 (37.3)
Drug	
Anticancer drug	81 (60.4)
Opioid	58 (43.2)
Body mass index (kg/m^2^)	18.9 <16.8-21.3>
	N = 130 (due to missing data)
Hemoglobin (g/dL)	10.5 <9.2-12.2>
Albumin (g/dL)	3.2 <2.8-3.5>
C-reactive protein (mg/dL)	1.35 <0.24-4.63>
Number of applicable prescribing criteria	
0	13 (9.7)
1	35 (26.1)
2	42 (31.4)
3	44 (32.8)
Glasgow prognostic score	
0	27 (20.2)
1	37 (27.6)
2	70 (52.2)
Prognostic nutritional index	37.4 <33.0-41.9>

Investigation of the timing of anamorelin initiation

**Figure 2:**
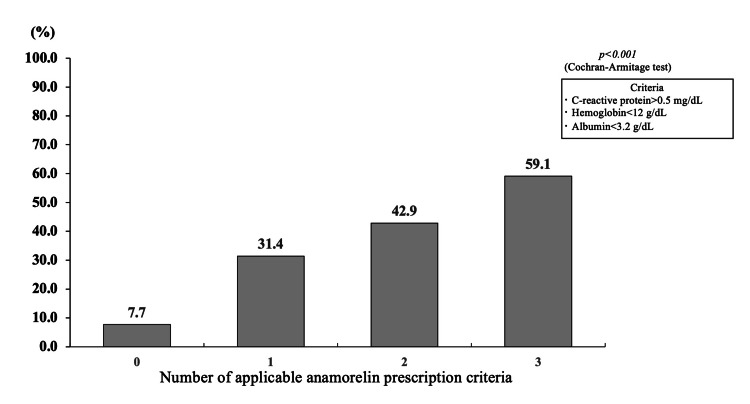
Number of applicable prescribing criteria (CRP >0.5 mg/dL, hemoglobin<12 g/dL, albumin <3.2 g/dL) for anamorelin and percentage of patients who died in less than three months after starting anamorelin therapy (n = 134) Number of patients n (%) for each category: 0: 13 (7.7%), 1: 35 (31.4%), 2: 42 (42.9%), 3: 44 (59.1%). CRP: C-reactive protein.

**Figure 3:**
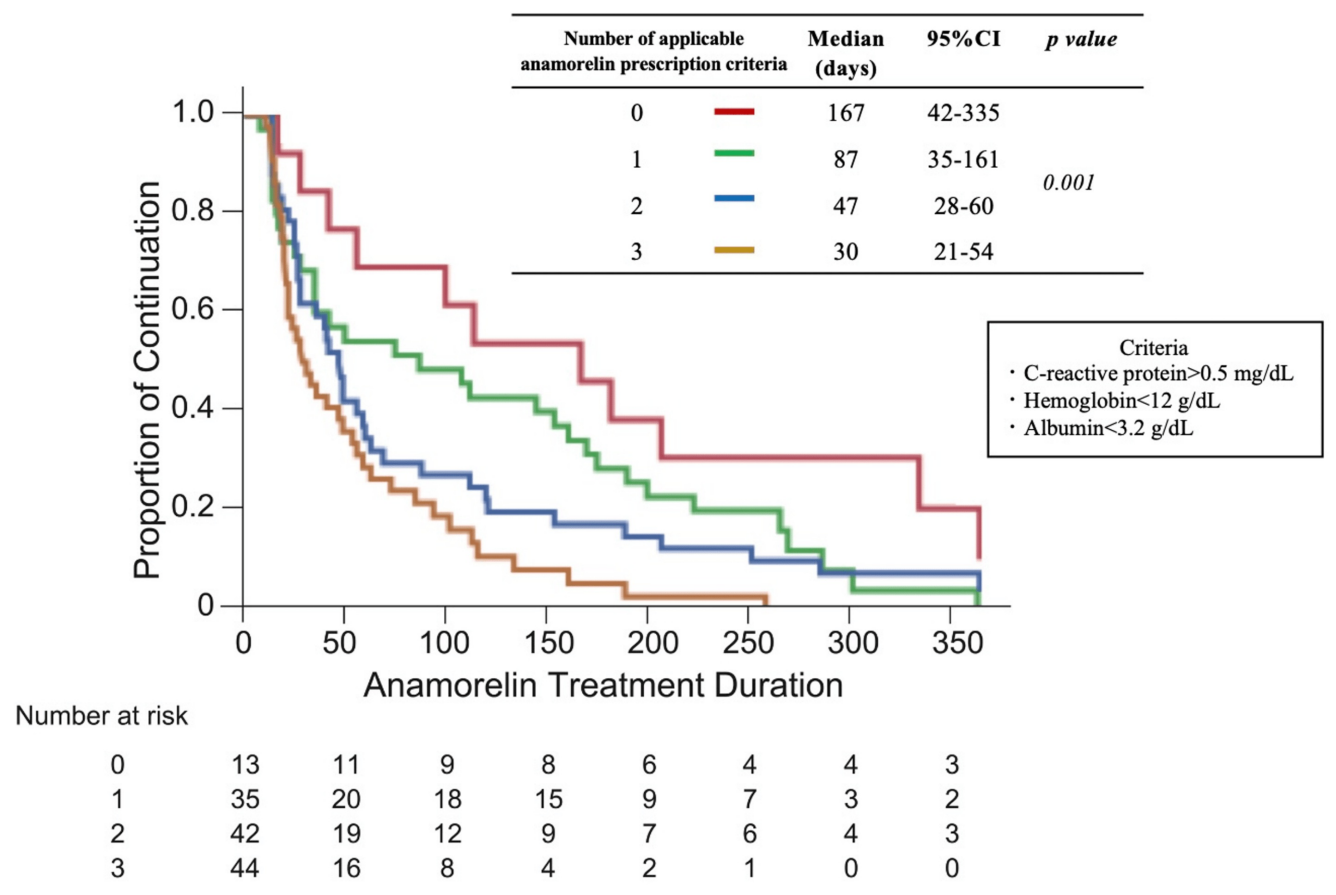
Number of anamorelin treatment criteria and duration of anamorelin treatment CI: confidence interval.

**Figure 4:**
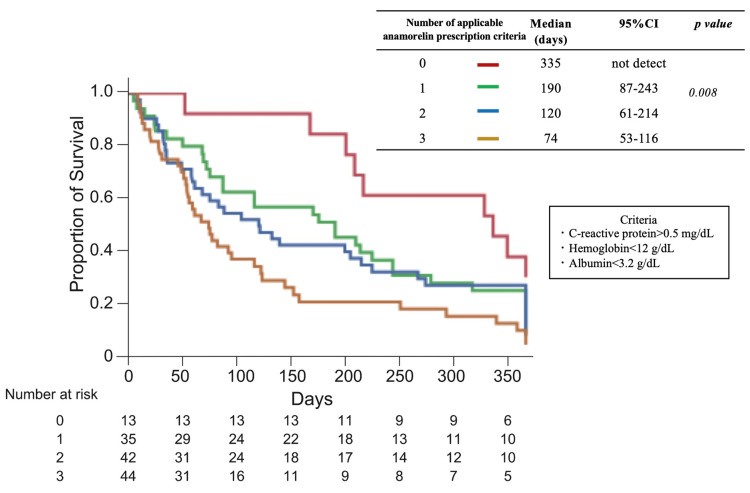
Number of criteria for anamorelin treatment and survival after initiation of anamorelin treatment CI: confidence interval.

To determine the stage of cachexia in the study patients, the percentage of patients who met one of the definitions of refractory cachexia, “survival of less than 3 months,” was examined. 41.8% of patients were confirmed to have died within three months of starting treatment. The proportion of patients who died in less than three months significantly increased with the number of objective prescribing criteria (p < 0.001) (Figure 2). The number of objective prescribing criteria was considered to be a key factor in considering when to start anamorelin treatment, so we investigated the number of criteria, duration of anamorelin treatment, and survival time. The median duration of anamorelin treatment (0 vs. 1 vs. 2 vs. 3) was 167 vs. 87 vs. 47 vs. 30 days (Figure 3), and the median survival time was 335 vs. 190 vs. 120 vs. 74 days (Figure 4).

Factors for continuation of anamorelin treatment

**Figure 5:**
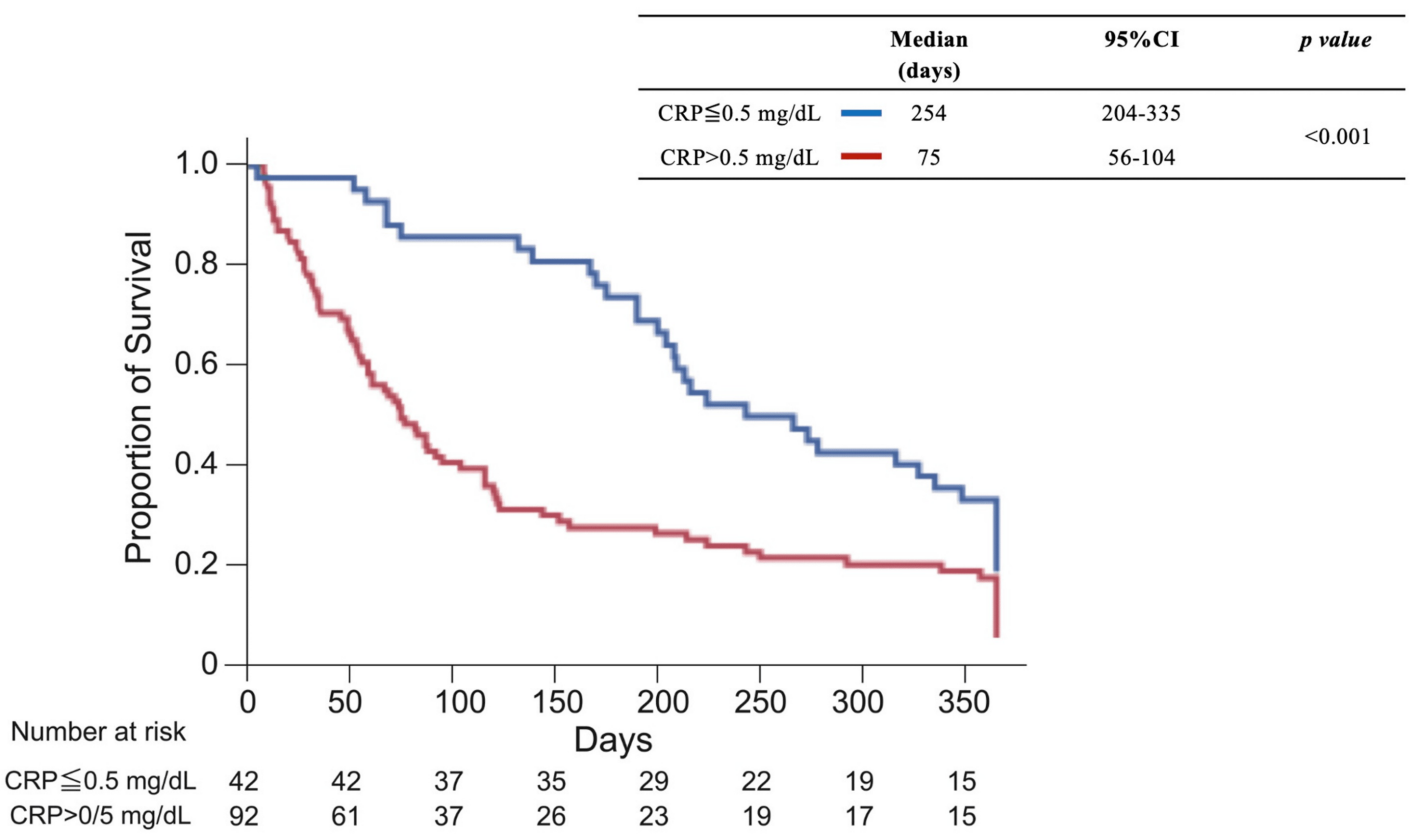
Survival time after initiation of anamorelin therapy (CRP ≤ 0.5 mg/dL vs. CRP > 0.5 mg/dL) CI: confidence interval; CRP: C-reactive protein.

We analyzed the factors related to continuation of anamorelin treatment in a multivariate analysis, considering that treatment efficacy is poor at the stage of defining refractory cachexia and that treatment intervention should be performed at an earlier stage of cachexia. We defined continuation of anamorelin treatment as 12 weeks or longer, based on previous clinical studies [6,7]. As a result, CRP >0.5 mg/dL was extracted as a factor (Table 2). Based on this, patients were divided into two groups: CRP ≤0.5 mg/dL and CRP >0.5 mg/dL. Patient backgrounds are shown in Table 3. Albumin levels were significantly higher, and CRP was lower in the CRP ≤0.5 mg/dL group. The CRP ≤0.5 mg/dL group had a larger proportion of patients treated with anticancer agents and a smaller proportion of patients using medical narcotics. Comparing the CRP ≤0.5 mg/dL and CRP >0.5 mg/dL groups, the median number of anamorelin treatment days was 137 (42-223) vs. 32 (19-82) days. The CRP ≤0.5 mg/dL group had a significantly greater proportion of patients with improved symptoms of appetite and fatigue, with a median survival of 254 days (95% confidence interval [CI]: 204-335 days), significantly longer (p < 0.001) than the CRP >0.5 mg/dL group (75 days [95% CI: 56-104 days]) (Figure 5).

**Table 2:** Factors associated with continuation of anamorelin treatment for 12 weeks by multivariate analysis CI: confidence interval.

Factor	Multivariate analysis		
	OR	95% CI	p-Value
Hemoglobin (<12 vs. ≥12)	1.34	0.30-1.76	0.507
Albumin (<3.2 vs. ≥3.2)	2.07	0.89-4.83	0.087
C-reactive protein (>0.5 vs. ≤0.5)	4.05	1.76-9.62	<0.001

**Table 3:** Patient background characteristics and anamorelin treatment duration and symptom improvement CRP: C-reactive protein; IQR: interquartile range. a) Chi-square test, b) Mann-Whitney U test.

Background characteristics	CRP ≤0.5 mg/dL (N = 42); n (%), median ＜IQR＞	CRP >0.5 mg/dL (N = 92); n (%), median ＜IQR＞	p-Value	Statistical tests
Sex				
Male	25 (59.5)	61 (66.3)	0.435	a)
Female	17 (40.5)	31 (33.7)		
Age	70 <57-76>	72 <65-78>	0.120	b)
Primary site of cancer				
Gastric	15 (35.7)	17 (18.5)	0.871	a)
Colorectum	7 (16.7)	17 (18.5)		
Pancreas	10 (23.8)	18 (19.5)		
Lung (non-small cell lung cancer)	10 (23.8)	40 (43.5)		
Drug				
Anticancer drug	39 (92.9)	42 (45.7)	<0.001	a)
Opioid	6 (14.3)	52 (56.5)	<0.001	a)
Body mass index (kg/m^2^)	18.0 <16.8-21.4>	19.0 <16.9-21.3>	0.488	b)
	N = 42	N = 88		
Hemoglobin (g/dL)	11.1 <9.7-12.6>	10.0 <8.9-11.9>	0.05	b)
Albumin (g/dL)	3.6 <3.3-3.9>	3.1 <2.7-3.3>	<0.001	b)
C-reactive protein (mg/dL)	0.12 <0.08-0.19>	2.97 <1.26-6.79>	<0.001	b)
Number of applicable prescribing criteria				
0	13 (31.0)	0 (0)	<0.001	a)
1	21 (50.0)	14 (15.2)		
2	8 (19.0)	34 (37.0)		
3	0 (0)	44 (47.8)		
Prognostic nutritional index	41.7 <37.7-46.0>	35.9 <31.2-9.3>	<0.001	b)
Anamorelin treatment duration and symptom improvement				
Treatment duration (days)	137 <42-223>	32 <19-82>	<0.001	b)
Symptom improvement	32 (76.2)	50 (54.3)	0.002	a)

Status after 12 weeks of anamorelin treatment

Table 4 shows the treatment status and reasons for discontinuation of medication after 12 weeks of anamorelin treatment. The number of patients who were on anticancer therapy after 12 weeks of anamorelin treatment was 73.8% in the CRP ≤0.5 mg/dL group and 27.2% in the CRP >0.5 mg/dL group, significantly higher in the CRP ≤0.5 mg/dL group (p < 0.001). Death occurred in 14.3% of patients in the CRP ≤0.5 mg/dL group and 55.4% in the CRP >0.5 mg/dL group, significantly higher in the CRP >0.5 mg/dL group (p < 0.001). In the CRP >0.5 mg/dL group, 21.7% of patients discontinued anamorelin within 12 weeks due to death, significantly more than in the CRP ≤0.5 mg/dL group (p < 0.01).

**Table 4:** Treatment status after anamorelin administration and reasons for discontinuation a) Chi-square test. CRP: C-reactive protein.

	CRP ≤0.5 mg/dL (N = 42); n (%)	CRP >0.5 mg/dL (N = 92); n (%)	p-Value	Statistical tests
Treatment status after 12 weeks of anamorelin administration				
Chemotherapy	31 (73.8)	25 (27.2)	<0.001	a)
Supportive palliation	5 (11.9)	16 (17.4)	0.417	a)
Died	6 (14.3)	51 (55.4)	<0.001	a)
Reasons for discontinuation of anamorelin				
Treated for 12 weeks	26 (61.9)	22 (23.9)	<0.001	a)
Adverse events	2 (4.8)	9 (9.8)	0.326	a)
No effect	6 (14.2)	21 (22.8)	0.252	a)
Died	1 (2.4)	20 (21.7)	0.004	a)
Patient requested to discontinue	2 (4.8)	2 (2.2)	0.414	a)
Changing hospital	0 (0)	2 (2.2)	0.335	a)
Others	5 (11.9)	9 (9.8)	0.709	a)
Unknown	0 (0)	7 (7.6)	0.066	a)

## Discussion

In this study, we investigated the timing of initiation of anamorelin treatment in cancer treatment patients with cachexia. 41.8% of the subjects died within three months of starting anamorelin treatment. In a report by Tsukiyama et al. [9], 42.5% of patients died within 100 days of starting anamorelin administration, a result similar to that of this study. In the present study, 52.2% of patients had a GPS score of 2. GPS classification is a prognostic factor independent of cancer stage [10] and has been reported to be an appropriate indicator of cachexia [11]. In addition, a GPS score of 2 with weight loss has been reported as refractory cachexia [12], and patients with a GPS score of 2 are considered to be close to refractory cachexia. Focusing on the duration of anamorelin administration, in clinical trials, approximately 60% of patients were able to complete 12 weeks of anamorelin administration, but in the interim report of anamorelin PMS, only approximately 30% of patients continued to take the medication for 7-12 weeks or more. A survey of Japanese physicians and healthcare professionals found that 62.7% of respondents regarded “cancer cachexia” as a terminal-stage symptom of cancer [13]. Consequently, anamorelin, a drug used to treat cachexia, may be initiated in Japan when symptoms are considered indicative of the terminal stage, such as severe emaciation or prolonged bed rest. In addition, essential requirements for eligibility for anamorelin treatment include weight loss of 5% or more within six months and loss of appetite, but a recent survey has reported that doctors become aware of 73.8% of cases of loss of appetite and 37.9% of cases of weight loss through patient complaints [14]. This means that medical professionals are unlikely to notice these symptoms, and by the time a patient complains, the stage of cachexia treatment may have already been missed. Furthermore, the objective assessment criteria for prescribing anamorelin, CRP >0.5 mg/dL or hemoglobin <12 g/dL or albumin<3.2 g/dL, often do not reveal changes in physical findings, making it difficult to detect cachexia and potentially delaying the treatment of cachexia. Furthermore, in an international survey of healthcare providers on cancer cachexia, when asked about the weight loss rate of cancer patients that they define as "cachexia," 29.1% of medical professionals answered "weight loss of more than 5%," 28.8% answered "weight loss of more than 10-20%," and 17.6% answered "there is no consistent definition of cachexia." In addition, as a reason why clinicians do not regularly screen for cachexia, 38.8% answered "there are no standard tools or instruments to screen patients for cachexia" [15]. Therefore, the difficulty of diagnosing cachexia may be a factor in delaying treatment. Furthermore, in the same survey, 52.7% of respondents thought that patients should be screened for weight loss at the time of cancer diagnosis, and 91.5% thought that weight loss should be monitored all or most of the time during treatment. On the other hand, 47.4% of respondents said they measure weight at each appointment, and 61.9% said it is necessary to track weight over time. "A 5% weight loss within 6 months" is an indication for anamorelin treatment, and Fearon et al. have reported it to be one of the diagnostic criteria for cachexia [1]. Therefore, measuring body weight at each consultation and identifying weight loss at an early stage may lead to the success of anamorelin treatment. Interestingly, the fewer the number of criteria that meet the prescription criteria of CRP >0.5 mg/dL, hemoglobin <12 g/dL, and albumin <3.2 g/dL, the significantly lower the proportion of patients who died within less than three months of starting anamorelin treatment. There was also anamorelin treatment duration and survival period tended to be longer. Since clinical trials showed an increase in lean body mass after 12 weeks of anamorelin treatment [6,7], we investigated factors related to whether or not the anamorelin treatment period was 12 weeks, and CRP was extracted in multivariate analysis. Furthermore, when patients were divided into two groups based on their CRP levels at the time of starting anamorelin treatment (patients with CRP ≤0.5 mg/dL and patients with CRP >0.5 mg/dL), it was found that the CRP ≤0.5 mg/dL group had a higher proportion of patients receiving chemotherapy and a lower proportion of patients using medical narcotics. Additionally, the percentage of patients meeting two or more prescribing criteria was 19% in the CRP ≤0.5 mg/dL group and 84.8% in the CRP >0.5 mg/dL group, suggesting that these criteria (hemoglobin, albumin, CRP) may include items that are early indicators of treatment eligibility. Cancer cachexia is associated with acute-phase response proteins [16,17], and CRP has been reported to be a sensitive marker for diagnosing cancer cachexia [18]. Since the half-life of CRP is known to be 19 hours, careful monitoring of CRP levels and initiation of drug therapy may lead to early introduction of anamorelin in patients with cancer cachexia. Furthermore, when comparing the CRP ≤0.5 mg/dL group and the CRP >0.5 mg/dL group, it was found that the duration of anamorelin treatment and the survival period were significantly longer in the CRP ≤0.5 mg/dL group. Additionally, at the start of anamorelin treatment and 12 weeks after administration, the number of patients undergoing chemotherapy and the continuation rates were 39/32/82% for the CRP ≤0.5 mg/dL group and 42/25/55% for the CRP >0.5 mg/dL group. It can be inferred that at the start of anamorelin treatment, the proportion of patients undergoing chemotherapy was significantly higher in the CRP ≤0.5 mg/dL group, which may influence the extension of survival due to the suppression of tumor growth. However, the high rate of chemotherapy continuation suggests that anamorelin treatment may have influenced chemotherapy continuation and contributed to extending survival. Based on these findings, careful monitoring of CRP, a dynamic indicator, is important during cancer treatment, and if any of the prescription criteria are met, consideration of administering anamorelin could be given, which could be introduced early, even during cachexia, and patients may benefit from anamorelin treatment. There have been several reports of studies showing the effectiveness of anamorelin in clinical practices. 1) Those who had less weight loss before anamorelin administration showed improved weight after anamorelin administration [19]; 2) when the CRP-albumin ratio (CAR) was low at the time of anamorelin administration, weight after anamorelin administration was maintained or increased [20]; 3) when total protein, albumin, transferrin, and PNI were high at the time of anamorelin administration, lean body mass was maintained or increased [21]; and 4) starting anamorelin treatment when neutrophil-to-lymphocyte ratio is less than 4.4 is likely to improve outcomes [22]. All of the reports indicate that the early introduction of anamorelin in good condition, even in cases of cachexia, is effective. This study has several limitations. First, this study included only anamorelin-treated patients and lacked a control group. Second, it is a single-center retrospective study, which is subject to selection bias. Third, there were few objective numerical records of changes in calorie intake, body weight, and lean body mass after anamorelin administration. The effect of anamorelin is an increase in lean body mass, and the extension of survival time is considered to be a by-product of this. Since various factors affect the extension of survival time, it is difficult to determine whether starting cachexia treatment early contributes to the extension of survival time. Considering these factors, a prospective multicenter study is necessary to investigate changes in diet, body weight, and lean body mass with anamorelin administration. In addition, the limitations, potential confounders, and the need for further prospective studies to validate the results should be discussed. Since cachexia is a complex disease concept influenced by multiple factors, recognizing its diagnosis in the absence of an obvious cause of elevated CRP is useful. However, further biomarker discovery is desirable. Nevertheless, in daily clinical practice, it receives the impression that prescriptions are continued not only for weight gain but also for improvement of appetite and patient satisfaction.

## Conclusions

It was suggested that in clinical practice, there may be a delay in initiating anamorelin treatment for patients with cancer cachexia. The importance of carefully monitoring CRP and considering anamorelin initiation at the appropriate time became apparent. This may allow for early introduction of anamorelin therapy that may be beneficial in the treatment of cancer cachexia.
